# Reduced Reelin Expression in the Hippocampus after Traumatic Brain Injury

**DOI:** 10.3390/biom10070975

**Published:** 2020-06-29

**Authors:** Valentina Dal Pozzo, Beth Crowell, Nicholas Briski, David P. Crockett, Gabriella D’Arcangelo

**Affiliations:** 1 Graduate Program in Neuroscience, Rutgers Robert Wood Johnson Medical School, Piscataway, NJ 08854, USA; valentinadalpozzo@gmail.com; 2 Department of Cell Biology and Neuroscience, Rutgers, the State University of New Jersey, Piscataway, NJ 08854, USA; bcrowell@dls.rutgers.edu (B.C.); ndb55@scarletmail.rutgers.edu (N.B.); 3 Department of Neuroscience and Cell Biology, Rutgers Robert Wood Johnson Medical School, Piscataway, NJ 08854, USA; crockett@rwjms.rutgers.edu

**Keywords:** hippocampus, cerebral cortex, cell death, trauma

## Abstract

Traumatic brain injury (TBI) is a relatively common occurrence following accidents or violence, and often results in long-term cognitive or motor disability. Despite the high health cost associated with this type of injury, presently there are no effective treatments for many neurological symptoms resulting from TBI. This is due in part to our limited understanding of the mechanisms underlying brain dysfunction after injury. In this study, we used the mouse controlled cortical impact (CCI) model to investigate the effects of TBI, and focused on Reelin, an extracellular protein that critically regulates brain development and modulates synaptic activity in the adult brain. We found that Reelin expression decreases in forebrain regions after TBI, and that the number of Reelin-expressing cells decrease specifically in the hippocampus, an area of the brain that plays an important role in learning and memory. We also conducted in vitro experiments using mouse neuronal cultures and discovered that Reelin protects hippocampal neuronal cells from glutamate-induced neurotoxicity, a well-known secondary effect of TBI. Together our findings suggest that the loss of Reelin expression may contribute to neuronal death in the hippocampus after TBI, and raise the possibility that increasing Reelin levels or signaling activity may promote functional recovery.

## 1. Introduction

Traumatic brain injury (TBI) is a relatively common problem often associated with accidents or violent sport activities, leading to severe neurological dysfunction or death. It is estimated that 10 million people will be affected annually by 2020. Thus, TBI is a major cause of death and disability [1,2]. The long-term clinical symptoms caused by TBI are varied, but often include memory loss, headaches, speech difficulties, cognitive abnormalities, epilepsy, and motor deficits like weakness and loss of balance [3].

TBI produces two types of tissue responses, classified as primary or secondary damage. The primary damage is the direct result of the mechanical insult to the brain tissue, whereas the secondary damage develops over an extended period of time, indirectly as a result of the altered biochemical environment [4]. The initial response of the brain to the primary damage is an inflammatory reaction mediated by microglia and astrocytes that regulate the recruitment of leukocytes and chemokines to the injured brain [5]. Secondarily, there is an increase in membrane permeability that leads to membrane depolarization and excessive release of neurotransmitters such as the excitatory neurotransmitter glutamate. The increased release of glutamate disrupts the Na^+^/K^+^/Ca^2+^ flux and leads to the overstimulation of N-methyl-D-aspartate (NMDA) receptors and α-amino-3-hydroxy-5-methyl-4 isoxazolepropionic acid (AMPA) receptors [6,7]. This in turn can lead to neuronal dysfunction and cell death (excitotoxicity).

Although animal models cannot completely mimic the injury to the human brain, they are essential for understanding the molecular and cellular events that occur after TBI and for developing new treatments [8]. The controlled cortical impact (CCI) is a common experimental model of TBI that uses a pneumatic impact device to deliver an injury to the exposed intact dura [9]. This model confers reproducible injuries, and reproduces many aspects of human TBI such as cortical tissue loss, acute subdural hematoma, axonal injury, and blood–brain barrier dysfunction [10,11,12]. Depending on the severity of the impact, this type of injury can result in neuroinflammation, cortical, hippocampal, and thalamic degeneration [13]. The CCI model also produces many neurobehavioral deficits common in human TBI patients, including long-term cognitive and motor problems [14,15].

Despite the high impact of TBI on society, there are currently no effective pharmacological treatments to ameliorate or prevent the development of long-term brain dysfunction, and little is known about the molecular basis of cognitive loss and functional recovery. A current trend in TBI research is to investigate mechanisms that would help to repair the neuronal circuitry after brain damage and to re-establish synaptic connections and network functions. Proteins that are involved in restoring the integrity of cellular systems may thus play an important role in functional recovery. In this study, we focused on Reelin, a protein initially discovered as a major regulator of mammalian brain development, controlling the formation of laminated cortical structures [16]. Complete loss of Reelin in the spontaneous neurological mutant mouse *reeler* results in neuronal ectopia due to defective radial migration during embryonic brain development [17]. We now know that Reelin is also important for postnatal brain maturation and adult brain function, promoting dendrite outgrowth, synapse formation, and synaptic plasticity [18]. Most, if not all, of these functions are mediated by a signaling pathway including two cell-surface receptors of the lipoprotein receptor superfamily, the apolipoprotein E receptor-2 (ApoER2) and the very low-density lipoprotein receptor (VLDLR), Src-family kinases (SFKs, mainly Fyn and Src) the intracellular adapter protein Dab1, and downstream signaling kinases, such as Akt, and GSK3β [19]. Adult-specific inducible Reelin knock out mice performed normally in cognitive tasks, suggesting that this protein is not essential for brain function under normal conditions [20]. These mice, however, showed increased susceptibility to Aβ-induced synaptic suppression, suggesting that Reelin may protect the adult brain from degeneration or dysfunction under disease conditions.

The link between Reelin in TBI has not been previously investigated. Here, we used the CCI model to investigate whether Reelin expression is altered by brain trauma in vivo, and dissociated neuronal cultures to investigate its potential role in neuroprotection after glutamate-induced excitotoxicity. Our data reveal the long-term loss of Reelin expression after TBI specifically in the hippocampus, and a novel function of Reelin in promoting the survival of hippocampal neurons.

## 2. Materials and Methods

### 2.1. Animal Handling

Animals used in this study were handled in accordance with a protocol approved by the Association for Assessment and Accreditation of Laboratory Animal Care (AAALAC) committee at Rutgers, the State University of New Jersey. For all experiments, mice of the CD-1 strain were purchased from Charles River Laboratory (Wilmington, MA. USA).

### 2.2. Controlled Cortical Impact (CCI) Injury Model

To prepare the mice for CCI injury or sham craniectomy surgery, young adult mice (1–2 month-old) were anesthetized with 4–5% of isoflurane in 100% O_2_ and received buprenorphine (0.1 mg/kg) intraperitoneally as preemptive analgesia. The mice were then placed in a stereotaxis frame equipped with a micromanipulator (Kopf Instruments, Tujunga, CA, USA), the isoflurane flow was maintained at 2%, and the animals were monitored during the entire procedure. An incision was made in the middle from the eyes to the neck and a topical anesthetic was applied on the skull (bupivacaine, 0.025% in saline). A craniectomy was made above the right hemisphere, halfway between bregma and lambda, with a 2.7 mm diameter trephine to remove a piece of the skull just above the parietal cerebral cortex. The dura was kept intact and animals which showed herniation or dura damage were discarded. Under microscopic control, the PinPoint Precision Cortical Impactor, Model PCI3000 (Hatteras Instruments, Cary, NC, USA) was positioned over the exposed dura, tilted at a 4–10° angle to ensure that the entire surface of the probe was in contact with the dura mater. The CCI injury was delivered with the following parameters: 1.5 mm depth, 3 m/s velocity and 500 ms contusion time. Using these parameters, the impactor penetrates the cerebral cortex, causing extensive structural damage in the surrounding region, but does not penetrate or cause apparent tissue damage in the underlying hippocampal formation. After delivering the brain injury the skin was closed with Vetbond tissue adhesive (3M) and the mice received an intraperitoneal saline injection before being allowed to recover in their home cage.

### 2.3. Brain Tissue Section Preparation

Mice were sacrificed at different time points after injury. For immunofluorescence experiments they were anesthetized with Avertin (>300 mg/kg) and perfused transcardially with phosphate-buffered saline solution (PBS) followed by 4% paraformaldehyde (PFA) in PBS. The brains were dissected, post-fixed in 4% PFA for 4 h at 4 °C, and cryoprotected by incubation at 4 °C in a 30% sucrose solution in PBS. Brains were mounted onto a sliding microtome using Tissue-Tek OCT (Sakura USA, Torrance, CA, USA) and sectioned (30 µm sections) for histology on glass slides.

### 2.4. Immunofluorescence Assays

To analyze Reelin-expressing cells, three different sections/mouse were obtained at approximately the −1.5, −2.0, and −2.50 mm distance from bregma. Brain sections were washed in PBS for 5 min two times to remove the OCT from the slides. Then the slides were permeabilized with 0.1% Triton X-100 in PBS for 20 min and incubated with blocking buffer (5% normal goat serum in 0.1% Triton-X-100 in PBS) for 1 h. The sections were incubated overnight at 4 °C with Alexa-Fluor 488-conjugated mouse monoclonal anti-Reelin antibodies (clone G10) (MAB5364A4, Millipore, Burlington, MA, USA) diluted 1:500 in blocking buffer, and then washed with PBS (3 × 5 min). Coverslips were mounted with Vectashield Mounting Medium containing DAPI (Vector Laboratories, Burlingame, CA, USA). Sections were imaged using a LSM800 confocal microscope (Zeiss, Oberkochen, Germany) and the number of Reelin+ cells was manually determined in different brain regions such as the hippocampus proper or dentate gyrus. For the cerebral cortex we calculated the density of Reelin+ cells by dividing the number of cells by the total area of six regions of interest (ROIs).

### 2.5. mRNA Isolation and Quantitative Reverse Transcription PCR (RT-qPCR) Analysis

Fresh cortical tissue close to the injury area, and hippocampal tissue were collected in the ipsilateral and contralateral side of the brain at different time points after TBI (24 and 72 h). The total RNA was purified using the Qiagen (Hilden, Germany) RNeasy Kit and transcribed into cDNA using a High-Capacity cDNA Reverse Transcription kit (Applied Biosystems, Foster City, CA, USA). The resulting cDNA was analyzed by RT-qPCR using the Power SYBR Green master mix (Applied Biosystems, Foster City, CA, USA). The expression level of specific genes was analyzed using Applied Biosystems Real-Time PCR machines (Table 1). *Reelin*, *CXCL10*, and *GFAP* expression was analyzed using the StepOne System and the StepOne Software v2.3, whereas *Gad1*, *Parvalbumin*, *Somatostatin*, and *Map2* expression was analyzed using the QuantStudio 3 and the QuantStudio Design&Analysis V1.4.3 software. All gene expression values were first normalized to the ribosomal protein S12 (internal control), and then were further normalized to the average value of sham contralateral samples.

### 2.6. Reelin Protein Expression and Purification

Reelin-conditioned medium was collected as the supernatant of a stable mammalian cell line (CER) derived from 293-EBNA cells (Invitrogen, Carlsbad, CA, USA) that were transfected with a plasmid encoding the full length *Reelin* cDNA (pCER) cloned into the pCEP4 vector (Invitrogen, Carlsbad, CA, USA). Hygromycin B-resistant clones were selected and tested for *Reelin* expression, and then pooled to generate the CER cell line [26]. The cells were cultured in a serum free Neurobasal medium (Gibco Life Technologies, ThermoFisher, Waltham, MA, USA). The Reelin-conditional medium (CER) and the mock-control medium from the parental cell line (EBNA) were collected and stored at 4 °C with HEPES buffer (20 nM) (Gibco Life Technologies, ThermoFisher, Waltham, MA, USA) until use. Full length mouse Reelin protein was purified by affinity chromatography from transiently transfected mammalian cells according to an established protocol [27]. The highly concentrated purified protein was detected by SDS-PAGE followed by Coomassie staining, whereas the more diluted Reelin protein present in the CER medium was detected by SDS-PAGE (4–12% gradient gel, ThermoFisher, Waltham, MA, USA) followed by Western blot analysis using an unconjugated G10 Reelin mouse monoclonal antibody that was purified from hybridoma cell culture supernatants using Hi-Trap protein G columns (GE Healthcare, Chicago, IL, USA).

### 2.7. Dissociated Hippocampal Neurons

The hippocampus was dissected from the brain of embryonic day (E) 16 CD-1 mice, and cells were dissociated using Papain (Worthington, Columbus, OH, USA) in HBSS buffer with the supplement of 1M EDTA and 0.5 M CaCl_2_. Neurons were cultured in 24-well plates (1.0 × 10^5^ cells/well) coated with poly-L lysine in Neurobasal medium supplemented with 2% B-27 supplement and 0.5 mM L-glutamine (Invitrogen, Carlsbad, CA, USA).

### 2.8. Cell Death Staining and Analysis

After 10–13 days in vitro (DIV) hippocampal cells were pretreated with purified Reelin (50 nM) or CER medium for 30 min and then exposed to 30 µM of glutamate to induce excitotoxicity. EBNA mock medium was used as a control for CER. Then, 24 h after treatment, cells were stained with 10 μg/mL Propidium Iodide (PI) (ThermoFisher, Waltham, MA, USA) to label the nuclei of dead cells, and with Hoechst stain (1:1000, ThermoFisher, Waltham, MA, USA) to label all cell nuclei. Cultures were stained with these fluorescent dyes for 15 min in PBS at 37 °C, and then washed two times with PBS. Stained cells were imaged at the 10× magnification with an automated fluorescence microscope (INCell Analyzer 6000, GE Healthcare, Chicago, IL, USA). Multiple image fields (5–6 per well) were collected at random, and stained nuclei were manually counted in blind by two investigators. The percentage of co-labeled PI/Hoechst over total Hoechst-labeled nuclei was calculated from multiple wells in each treatment group as an index of cell death. 

### 2.9. Statistical Analysis

All data were statistically analyzed using the GraphPad (San Diego, CA, USA) Prism8 software. An alpha level set at *p* < 0.05 was set for statistically reliable rejection of the null hypothesis. Outliers identified with the ROUT test (Q = 1%) were eliminated from the analysis. The data were analyzed first for normal distribution using the Shapiro–Wilk test (=0.05). If the distribution was normal the values were further analyzed by paired *t*-tests comparing contralateral (C) and ipsilateral (I) values from the same mice, or ordinary one-way ANOVA followed by Tukey’s multiple comparison tests. If distribution was not normal, the data were analyzed using Wilcoxon matched-paired tests comparing contralateral (C) and ipsilateral (I) values from the same mice.

## 3. Results

### 3.1. Short-Term Effects of CCI on Inflammation, Reelin, and Neuronal Markers Expression in the Mouse Forebrain

In order to investigate the molecular events following TBI we used the CCI model to deliver a moderate injury to the mouse brain. We also included in this experimental set a group of mice that received craniectomy alone (sham).

**Figure 1:**
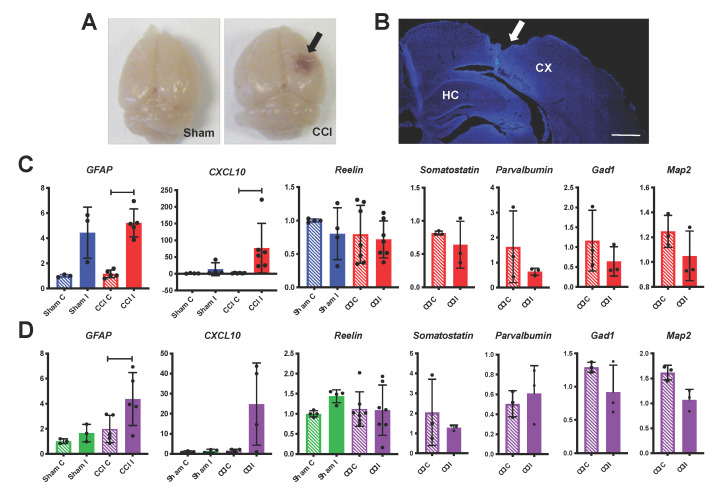
Inflammation but no change in Reelin or other neuron marker expression at 24 h post-controlled cortical impact (CCI). (**A**) Representative images of the whole brain from mice subjected to sham surgery or CCI. The cortical injury is clearly detectable in the ipsilateral side (I) of the CCI mouse (arrow). (**B**) Representative image of a brain section stained with the DAPI nuclear stain, showing extensive cortical damage in the side ipsilateral to the injury (arrow). CX = cerebral cortex; HC = hippocampus; scale bar = 500 μm. (**C**,**D**), RT-qPCR analysis of gene expression in cerebral cortical (**C**) or hippocampal (**D**) samples from sham or CCI groups. The following number of mice were analyzed for each gene: *GFAP*, *CXCL10*, and *Reelin* (*n* = 5–7 CCI, *n* = 3–4 sham); *Somatostatin*, *Parvalbumin*, *Gad1*, and *Map2* (*n* = 3 CCI). Statistical analysis was conducted comparing contralateral (C) to ipsilateral (I) values in each sham or CCI group using paired *t*-tests (for normally distributed values) or Wilcoxon matched-pair tests (for values that were not normally distributed). Inflammatory markers *GFAP* and *CXCL10* were significantly elevated in cortical CCI I samples. These genes were also elevated in hippocampal CCI I samples, but only *GFAP* was significantly increased. *GFAP* was also elevated in cortical sham I samples, although the values did not reach statistical significance. All other genes analyzed were unaffected by CCI or sham surgery. Scatter dot plots show individual data points and the mean values +/- standard deviation (SD). * *p* < 0.05; ** *p* < 0.01.

A total of 24 h after CCI, the injury was readily observable in the ipsilateral hemisphere by visual examination of the dissected brain (Figure 1A). To further assess the impact of the injury on brain anatomy, we perfused some mice, dissected the fixed tissue, sectioned it in the coronal plane, and stained it with DAPI nuclear stain. Imaging of the ipsilateral hemisphere revealed that the CCI injury typically causes extensive damage in the dorsolateral cerebral cortex but does not apparently disrupt the hippocampal formation (Figure 1B). Therefore, we decided to analyze the two forebrain regions separately. A cohort of mice subjected to CCI or sham surgery was euthanized 24 h after injury, fresh brain tissues were collected and dissected into four samples groups (contralateral or ipsilateral cerebral cortex, and contralateral or ipsilateral hippocampus). We then extracted total mRNA from all sample groups and performed RT-qPCR to examine the expression of genes such as *GFAP* and *CXCL10*, which are well-known to be upregulated in astrocytes and function as markers of the neuroinflammatory response following TBI [28,29]. *GFAP* levels were indeed strongly and significantly elevated in the ipsilateral cortex and hippocampus of mice subjected to CCI, compared to contralateral tissues (Figure 1C,D). In addition, elevated levels of *GFAP* were detected in the sham ipsilateral cortex samples compared to the contralateral tissue, but appeared to be normal in the sham hippocampus (Figure 1C,D). Similarly, levels of *CXCL10* were significantly increased in the ipsilateral cortex of CCI mice and were also elevated in the ipsilateral CCI hippocampus compared to contralateral samples, although the difference was not statistically significant (Figure 1C,D). Together these results indicate that 24 h after injury there is substantial inflammatory response to CCI in both the cortex and hippocampus, and some inflammation is also present in the cortex as a result of the craniectomy alone.

Next, we performed RT-qPCR in the same sample groups to examine the expression of *Reelin* after TBI, and also investigate other interneuron markers that were previously reported to be downregulated after TBI [30]. We found that the levels of *Reelin* as well as *Somatostatin*, *Parvalbumin*, and *Gad1* (the gene encoding GAD67) mRNAs were unaltered by CCI in both the cortex and hippocampus (Figure 1C,D). We also measured the expression of *Map2*, a marker for all mature neurons, and found that it is also unaltered by the injury (Figure 1C,D). Together these data indicate that, despite extensive cortical tissue damage and inflammatory response in both the cortex and hippocampus, the expression of many neuronal genes including *Reelin* is not altered 24 h after injury.

**Figure 2:**
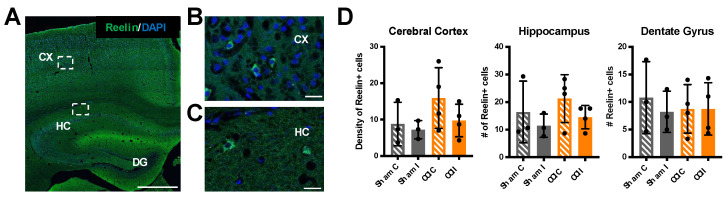
No change in the number of Reelin+ cells 24 h post-CCI. (**A**) Low magnification image of a CCI contralateral brain section immunolabeled with Alexa Fluor 488-conjugated Reelin monoclonal antibodies (green) and counterstained with DAPI (blue). (**B**) High-magnification image of the cerebral cortex (CX) corresponding to the inset in (**A**). (**C**) High-magnification image of the hippocampus (HC) corresponding to the inset in (**A**). CX = cerebral cortex; HC = hippocampus; DG = dentate gyrus. Scale bars: 500 μm (**A**) and 20 μm (**B** and **C**). (**D**) Quantification of Reelin+ cells from *n* = 3 sham and *n* = 4 CCI mice. Each value is the average of three different sections/brain. Data in the cerebral cortex represent the average density of Reelin+ cells (number of cells/six regions of interest (ROIs)), whereas data in the hippocampus and dentate gyrus represent the total number of cells counted in the entire structure. Statistical analysis was conducted comparing contralateral (C) to ipsilateral (I) values in each sham or CCI group using paired *t*-tests. The number of Reelin+ cells was not significantly affected by CCI or sham surgery. Scatter dot plots show individual data points and the mean values +/- SD.

To further examine Reelin expression after TBI we performed direct immunofluorescence on a different cohort of CCI and sham mice, 24 h after injury. Reelin-expressing cells were readily detected in both the neocortex and hippocampus using a fluorophore-conjugated monoclonal antibody. These cells correspond to a subset of forebrain interneurons, as previously described [31,32]. We obtained multiple tissue sections per each sham or CCI mouse and quantified the density of Reelin+ cells in the dorsolateral neocortex surrounding the site of injury, and the total number of Reelin+ cells in the hippocampus proper and in the dentate gyrus. The data show that there is no significant change in the number of Reelin+ cells in any of the regions examined 24 h after CCI, although the data from the cortex and hippocampus proper trended towards a decrease in the injured side (Figure 2).

### 3.2. Medium and Long-Term Effects of CCI on Reelin Expression in the Mouse Forebrain

**Figure 3:**
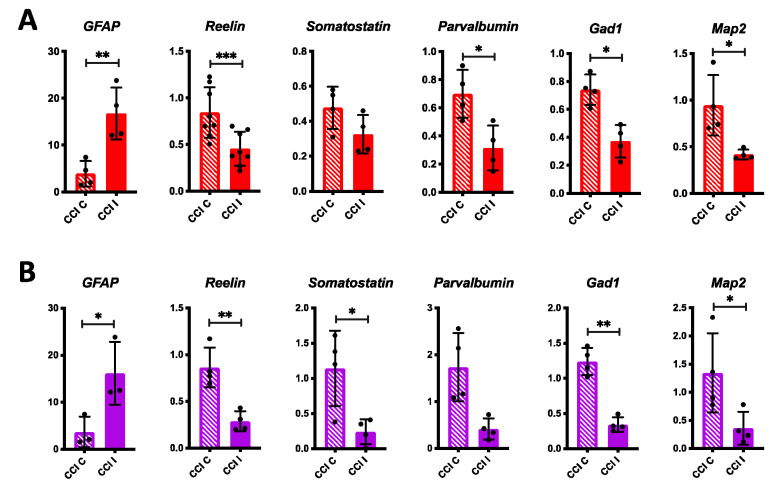
Decrease in *Reelin* and other neuron marker expression 72 h post-CCI. (**A**) RT-qPCR analysis of gene expression in cerebral cortical samples. (**B**) RT-qPCR analysis of gene expression in hippocampal samples. *n* = 3–8 CCI mice were analyzed for each gene. Statistical analysis was conducted comparing contralateral (C) to ipsilateral (I) values using paired *t*-tests. *GFAP* was significantly elevated in both cortical and hippocampal CCI I samples. *Reelin* levels were significantly downregulated in both cortical and hippocampal samples. All other genes analyzed were also downregulated by the injury in both cortical and hippocampal samples, although some values did not reach statistical significance. Scatter dot plots show individual data points and the mean values +/- SD. * *p* < 0.05, ** *p* < 0.01, *** *p* < 0.001.

To investigate potential long-term effects of CCI on Reelin expression we collected brain samples 72 h and 7 d after injury. At the 72 h time point, RT-qPCR analysis revealed persistent expression of *GFAP* in the ipsilateral cerebral cortex and hippocampus of CCI mice (Figure 3A,B). At this time, unlike the 24 h time point, we found that the levels of *Reelin* mRNA were significantly decreased in both, the ipsilateral cerebral cortex and the hippocampus (Figure 3A,B). Similarly, the expression of *Somatostatin*, *Parvalbumin*, *Gad1*, and *Map2* was consistently reduced in both the ipsilateral cerebral cortex and hippocampus of CCI mice, although the differences in cortical levels of *Somatostatin* and hippocampal levels of *Parvalbumin* did not reach statistical significance (Figure 3A,B). Our findings suggest that 72 h after CCI the forebrain retains an inflammatory response and downregulates the expression of many neuronal genes, including *Reelin*, which may contribute to cognitive dysfunction. This observed downregulation could result from the suppression of gene transcription mechanisms or from widespread neuronal cell death following TBI.

**Figure 4:**
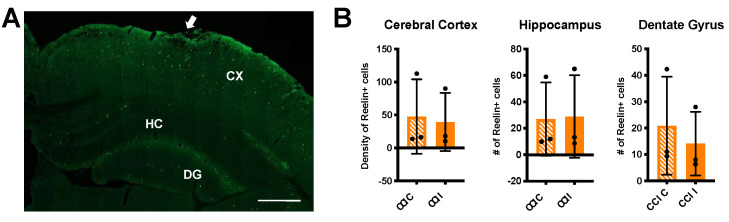
No change in the number of Reelin+ cells 72 h post-CCI. (**A**) Representative image of a CCI ipsilateral brain section immunolabeled with Alexa Fluor 488-conjugated Reelin monoclonal antibodies 72 h after injury. The arrow indicates the site of injury. CX = cerebral cortex; HC = hippocampus; DG = dentate gyrus. Scale bar: 500 μm. (**B**) Quantification of Reelin+ cells from *n* = 3 CCI mice. Each value is the average of three different sections/brain. Data in the cerebral cortex represent the average density of Reelin+ cells (number of cells/six ROIs), whereas data in the hippocampus and dentate gyrus represent the total number of cells counted in the entire structure. Statistical analysis was conducted comparing contralateral (C) to ipsilateral (I) values using Wilcoxon matched-pair tests (cerebral cortex) or paired *t*-tests (hippocampus and dentate gyrus). The number of Reelin+ cells was unaffected by CCI. Scatter dot plots show individual data points and the mean values +/- SD.

We also performed immunofluorescence at the 72 h time point to further examine Reelin protein expression (Figure 4A). We found no detectable loss of Reelin+ cells in the cerebral cortex or hippocampal formation at this time point (Figure 4B). Thus, 72 h post injury, a considerable decrease in *Reelin* gene expression was not accompanied by an appreciable loss of Reelin protein expression or death of Reelin-expressing cells in any of the forebrain regions examined.

**Figure 5:**
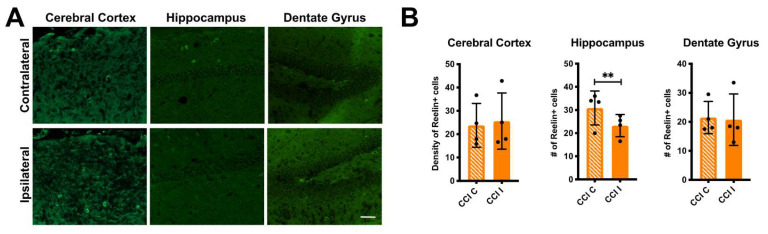
Loss of hippocampal Reelin+ cells 7d post-CCI. (**A**) Representative images of contralateral and ipsilateral brain sections immunolabeled with Alexa Fluor 488-conjugated Reelin monoclonal antibodies. The sections were obtained from a mouse subjected to CCI 7 d after injury. Scale bar: 50 μm. (**B**) Quantification of Reelin+ cells from *n* = 4 CCI mice. Each value is the average of 2–3 different sections/brain. Data in the cerebral cortex represent the average density of Reelin+ cells (number of cells/six ROIs), whereas data in the hippocampus and dentate gyrus represent the total number of cells counted in the entire structure. Statistical analysis was conducted comparing contralateral (C) to ipsilateral (I) values using paired *t*-tests. The number of Reelin+ cells was significantly reduced by CCI in the hippocampus proper, but not in the cerebral cortex or dentate gyrus. Scatter dot plots show individual data points and the mean values +/- SD. ** *p* < 0.01.

Finally, we analyzed Reelin-expressing cells by direct immunofluorescence 7 d post injury (Figure 5A). We found a significant and specific loss of Reelin+ cells in the ipsilateral hippocampus proper of CCI mice (Figure 5B). No changes were seen in the cerebral cortex or dentate gyrus. Apparently in the long-term, CCI uniquely affects the expression of Reelin protein or the survival of Reelin+ cells in the hippocampus proper.

### 3.3. Neuroprotective Effect of Reelin in Hippocampal Neurons

**Figure 6:**
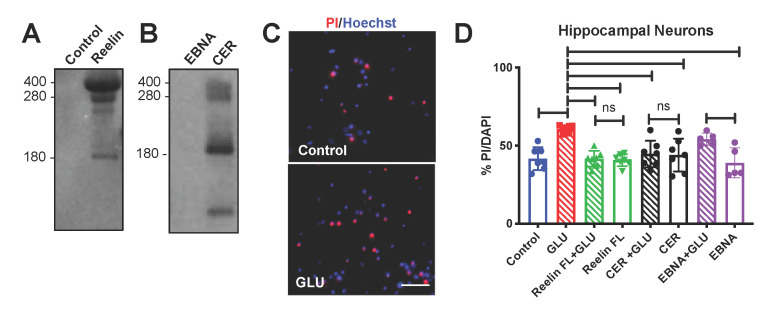
Reelin protects hippocampal neurons from glutamate-induced excitotoxicity. (**A**) Control purification buffer and purified Reelin protein were analyzed by SDS-PAGE and stained with Coomassie. Purified Reelin consists of a major band corresponding to the full-length protein (≈400 kDa), and proteolytic fragments. (**B**) Mock medium (EBNA) and Reelin conditioned medium (CER) were analyzed by Western blotting using G10 anti-Reelin antibodies. These antibodies detect a prominent band of ≈180 kDa corresponding to the major proteolytic N terminal fragment, in addition to the full-length protein and other cleavage products. (**C**) Representative images of hippocampal neurons treated overnight with control buffer or glutamate (GLU, 30μM), and stained with PI (red) and Hoechst (blue). The nuclei of dead cells appear red. Scale bar: 100 µm. (**D**) Quantification of cell death assays using days in vitro (DIV) 10–13 hippocampal neurons that were either untreated (control), treated with glutamate alone, or pretreated with purified Reelin (Reelin FL, 50 nM), Reelin conditioned medium (CER), or mock medium (EBNA) for 30 min and then exposed or not to GLU 30 μM for 24 h. The cells were stained with PI/Hoechst, random images where automatically acquired with the INCell Analyzer 6000, and the percentage of PI+ labeled nuclei was determined. Control values are significantly different only from GLU alone (blue asterisks), whereas GLU alone values are significantly different from all other groups except EBNA + GLU (red asterisks); there is no significant difference between Reelin FL + GLU and Reelin FL alone, or between CER + GLU and CER alone, but EBNA + GLU is significantly different from EBNA alone (purple asterisk). The data show that Reelin (purified or conditioned medium) protects hippocampal neurons from glutamate-induced excitotoxicity. Statistical analysis was conducted using one-way ANOVA with Tukey’s multiple comparison tests, *n* = 5–7 wells/group from three experiments (hippocampal neurons). Scatter dot plots show individual data points and the mean values +/- SD. * *p* < 0.05; ** *p* < 0.01; *** *p* < 0.001; **** *p* < 0.0001; ns = not significant.

The results above raised the possibility that hippocampal neurons may be particularly susceptible to cell death after injury, and that the loss of hippocampal Reelin after TBI may adversely affect the survival of neurons in this brain region. One of the best-known mechanisms underlying cell death after TBI is the transient increase in excitatory amino acids, such as glutamate, which causes widespread excitotoxicity. To determine whether Reelin can protect hippocampal cells from glutamate excitotoxicity we conducted a set of in-vitro experiments using neuronal cultures that were treated with Reelin prior to glutamate exposure. Two sources of recombinant Reelin were used in these experiments: purified protein and conditioned medium. Purified full-length Reelin was obtained as previously described [27], and the purification buffer was used as a control for this reagent. Reelin-conditioned medium was collected from a stably transfected cell line (CER) that was previously established in our laboratory [26]. Mock medium from a parental cell line (EBNA) was used as a control. Recombinant Reelin proteins were visualized by SDS-PAGE followed by Coomassie staining for the purified protein (Figure 6A), or by Western blot for the conditioned medium (Figure 6B). As expected, both these reagents contain in addition to full-length Reelin (≈400 kDa), several cleavage fragments generated by site-specific proteases [33,34,35]. We dissected hippocampi from embryonic mice, and cultured neurons for 10–13 days in vitro (DIV) in a serum-free medium using multi-well plates. Cultures were pretreated with purified Reelin or conditioned/mock medium for 30 min, and then exposed to 30 μM glutamate overnight. We then performed a cell death assay involving staining the cultures with propidium iodide (PI, a red fluorescent dye) to label the nuclei of dead cells, and Hoechst stain (blue fluorescent dye) to label all nuclei (Figure 6C). We used a high-content microscope to collect multiple random images per well, using multiple wells per treatment group, and measured the percentage of cell death under each condition. We performed three independent experiments and analyzed the pooled data. As expected, glutamate alone caused a significant increase in cell death, whereas purified Reelin, CER conditioned medium, or mock EBNA medium had no effect. Strikingly, Reelin and CER pretreatment caused a significant reduction in glutamate-induced cell death compared to the glutamate alone group (Figure 6D), demonstrating that Reelin protects hippocampal neurons from excitotoxicity. 

## 4. Discussion

In this work, we investigated the potentially novel link between Reelin and TBI using the CCI in vivo mouse model to examine Reelin expression, and an in vitro neuronal culture model to study the effect of Reelin on glutamate-induced excitotoxicity, a secondary effect of TBI. Using indirect immunofluorescence assays previous studies reported that Reelin expression is upregulated by TBI in two different lesion models: unilateral cortical ischemia and focal demyelination [36]. Reelin immunoreactivity was also reported to be increased in the mouse cornea and retina following ocular injury [37]. However, other groups reported cellular autofluorescence after TBI, and cautioned that this can hinder immunofluorescence analysis [38]. In addition, intact blood cells are known to be autofluorescent, and in fact, can be used to detect hemorrhages in postmortem studies [39]. Using indirect immunofluorescence with Reelin mouse monoclonal antibodies we also detected an artefactual signal specifically linked to the use of secondary anti-mouse IgG antibodies in damaged brain tissue (data not shown). It is conceivable that these secondary antibodies bind to endogenous immunoglobulin-like antigens expressed or exposed specifically in damaged brain cells. To circumvent this problem, in this study we used a commercially available fluorophore-conjugated mouse monoclonal antibody that enabled us to perform direct immunofluorescence assays, avoiding the use of secondary antibodies altogether. This fluorophore-conjugated Reelin monoclonal antibody (G10 clone) generated a strong and highly specific Reelin signal with virtually no background or artefactual signal even in damaged brain tissue. The results of this improved direct immunofluorescence approach were surprising and showed that Reelin expression was either unaffected or decreased after TBI, contrary to previous reports. Specifically, our direct immunofluorescence data demonstrated that the density of Reelin-positive cells was unaltered in the cerebral cortex at any time point examined, but it was reduced in the hippocampus at a late time point (7 days after TBI). The fact that other studies reported upregulation of Reelin expression after ischemia and demyelination in the corpus callosum could also be explained by differences in the injury model or examined brain region. Our findings that the number of Reelin-positive cells decrease in the hippocampus after CCI are consistent with a previous study that showed decreased Reelin protein levels after cerebral ischemia by Western blot analysis [40]. Interestingly, this study also showed that ischemia causes the upregulation of the microRNA miR-200c, which targets and suppresses *Reelin* gene expression, suggesting that this could contribute to stroke-induced injury. Reelin was also shown to be targeted by miR27b-3p, a microRNA that is elevated in cerebrospinal fluid (CSF), blood, and saliva of TBI patients [41]. These studies raise the intriguing possibility of targeting *Reelin*-suppressing microRNAs after TBI to prevent Reelin downregulation and improve clinical outcome.

Our immunofluorescence data revealed that brain injury causes a delayed loss of Reelin expression that specifically affects the hippocampus. These data are consistent with the literature, showing that GABAergic neurons are particularly susceptible to injury and become compromised by the increased glutamate excitability following TBI [30,42]. One study reported that calretinin-positive cells (a subpopulation of interneurons) exhibit reduced dendrite length 7 days post injury, concurrent with a reduction in the frequency of miniature inhibitory postsynaptic currents in layer V of the neocortex [43]. 

In the adult forebrain, Reelin is expressed mostly by inhibitory neurons [31,44]. A previous study showed that the number of inhibitory neurons positive for parvalbumin and somatostatin decreases 2–4 weeks after injury [30]. The results of our RT-qPCR are consistent with this finding and showed that *Reelin* and other inhibitory markers like *Gad1*, *parvalbumin*, and *somatostatin* were unaltered 24 h after TBI, but were significantly decreased in the injury side of the cortex and hippocampus 72 h post-injury. Additionally, most inhibitory markers, including *Reelin*, were reduced in the injured cortex at this later time point. These data suggested that TBI results in a global deficit of inhibitory neurons, which may alter connectivity and function in both the cortex and hippocampus. Indeed, one study provided evidence that the loss of inhibitory neurons following TBI leads to hyperexcitability and epilepsy [30]. However, we found that the expression of the pan-neuronal marker *Map2* was also reduced 72 h post-injury, suggesting that neuronal loss after TBI is not limited to interneurons. This widespread cell loss may relate to the activation of cell death mechanisms following the secondary effect of brain injury, including glutamate excitotoxicity. Our novel finding that Reelin expression is reduced in the forebrain after injury suggests that this event may contribute to cortical and hippocampal circuit dysfunction, leading to cognitive defects or memory loss after TBI. These observations also imply that recovery after TBI may be facilitated by molecules that can prevent Reelin downregulation or re-activate the Reelin signaling pathway. 

Our in vitro data indicate that Reelin reduces glutamate-induced excitotoxicity in hippocampal neurons. We found that the addition of Reelin to the medium of cultured hippocampal neurons exposed to glutamate significantly suppressed cells death. Reelin regulates the homeostasis of NMDAR in hippocampal neurons through SFK and Dab1 signaling [45,46,47]. It is possible that these receptors mediate the neuroprotective effect of Reelin in hippocampal neurons. Alternatively, the activation of signal transduction molecules commonly associated with cell survival, such as Akt, may be involved. Further studies are necessary to identify the molecular mechanisms that mediate neuroprotection by Reelin.

In conclusion, based on our findings, we reason that Reelin may play an important role in restoring the function of forebrain neuronal networks after TBI, and reducing the extent of glutamate-induced cell death in the hippocampus. This is an exciting and novel concept, however, it should be noted that the present study is limited due to the relatively small sample size utilized, particularly for in vivo TBI experiments. Thus, it will be important in the future to reproduce the present findings using a larger cohort of injured mice. It would also be interesting in the future to better understand the mechanisms that lead to the decrease in *Reelin* gene expression in both the cortex and hippocampus, and the specific loss of Reelin-positive cells in the hippocampus after TBI. Finally, it would be important to determine whether Reelin can protect hippocampal neurons from cell death in vivo, and investigate the molecular mechanisms involved in the neuroprotection from excitotoxicity. These studies would enable us to better envision how Reelin signaling could be exploited to facilitate functional recovery in TBI patients.

## Figures and Tables

**Table 1:** Primers used for RT-qPCR analysis. F = Forward. R= Reverse.

Gene	Sequence	Reference
*CXCL10*	F 5-ACCCAAGTGCTGCCGTCATT-3’ R 5-ATTCTCACTGGCCCGTCATC-3’	[21]
*Gad1*	F 5’-CGCTTGGCTTTGGAACCGACAA-3’ R 5’-GAATGCTCCGTAAACAGTCGTGC-3’	NM_008077
*GFAP*	F 5’-CGGGAGTCGGCCAGTTACCAG-3’ R 5’-TTTCCTGTAGGTGGCGATCTC-3’	[21]
*Map2*	F 5’-GCCAGCCTCGGAACAAACA-3’ R 5’-GCTCAGCGAATGAGGAAGGA-3’	[22]
*Reelin*	F 5’-CCCAGCCCAGACAGACAGTT-3’ R 3’-CCAGGTGATGCCATTGTTGA-3’	[23]
*Parvalbumin*	F 5’-TGTCGATGACAGACGTGCTC-3’ R 5’-TTCTTCAACCCCAATCTTGC-3’	[24]
*Somatostatin*	F 5’-TCTGCATCGTCCTGGCTTT-3’ R 5’-CTTGGCCAGTTCCTGTTTCC-3’	[25]
*S12*	F 5’-GGCATAGCTGCTGGAGGTGTAA-3’ R 5’-GGGCTTGGCGCTTGTCTAA-3’	[23]
